# Large area molybdenum disulphide- epitaxial graphene vertical Van der Waals heterostructures

**DOI:** 10.1038/srep26656

**Published:** 2016-06-01

**Authors:** Debora Pierucci, Hugo Henck, Carl H. Naylor, Haikel Sediri, Emmanuel Lhuillier, Adrian Balan, Julien E. Rault, Yannick J. Dappe, François Bertran, Patrick Le Fèvre, A. T. Charlie Johnson, Abdelkarim Ouerghi

**Affiliations:** 1 Laboratoire de Photonique et de Nanostructures (CNRS- LPN), Route de Nozay, 91460 Marcoussis, France; 2 Department of Physics and Astronomy, University of Pennsylvania, 209S 33rd Street, Philadelphia, Pennsylvania 19104, USA; 3 Institut des Nanosciences de Paris, UPMC, 4 place Jussieu, boîte courrier 840, 75252 Paris cedex 05, France; 4 Laboratoire d’Innovation en Chimie des Surfaces et Nanosciences, DSM/NIMBE/LICSEN (CNRS UMR 3685), CEA Saclay, 91191 Gif-sur-Yvette Cedex, France; 5 Synchrotron-SOLEIL, Saint-Aubin, BP48, F91192 Gif sur Yvette Cedex, France; 6 SPEC, CEA, CNRS, Universite Paris-Saclay, CEA Saclay, 91191 Gif-sur-Yvette Cedex, France

## Abstract

Two-dimensional layered transition metal dichalcogenides (TMDCs) show great potential for optoelectronic devices due to their electronic and optical properties. A metal-semiconductor interface, as epitaxial graphene - molybdenum disulfide (MoS_2_), is of great interest from the standpoint of fundamental science, as it constitutes an outstanding platform to investigate the interlayer interaction in van der Waals heterostructures. Here, we study large area MoS_2_-graphene-heterostructures formed by direct transfer of chemical-vapor deposited MoS_2_ layer onto epitaxial graphene/SiC. We show that via a direct transfer, which minimizes interface contamination, we can obtain high quality and homogeneous van der Waals heterostructures. Angle-resolved photoemission spectroscopy (ARPES) measurements combined with Density Functional Theory (DFT) calculations show that the transition from indirect to direct bandgap in monolayer MoS_2_ is maintained in these heterostructures due to the weak van der Waals interaction with epitaxial graphene. A downshift of the Raman 2D band of the graphene, an up shift of the A_1g_ peak of MoS_2_ and a significant photoluminescence quenching are observed for both monolayer and bilayer MoS_2_ as a result of charge transfer from MoS_2_ to epitaxial graphene under illumination. Our work provides a possible route to modify the thin film TDMCs photoluminescence properties via substrate engineering for future device design.

The study of graphene, a two dimensional (2D) atomic crystal formed of carbon atoms arranged in a honeycomb structure, is one of the hottest topics in material science due to its unique capabilities^1^,^2^. The importance of graphene not only lies in its properties but also on the fact that it opened the way and promoted the synthesis of many other 2D materials^3^. The last 10 years of research on graphene have led to many methods for synthesizing, transferring, manipulating and characterizing the properties of this 2D material, which can be applied to all layered van der Waals (vdW) materials. As one has full control of the 2D crystals, one can also create a stack of these crystals in a completely new heterostructures. Since the portfolio of the original 2D crystals is very rich^4^,^5^,^6^, a new world of materials is accessible. Combining different 2D layers with complementary characteristics can lead to new vdW heterostructures with tunable properties leading to an outstanding range of possible applications^7^,^8^. Among these systems, the combination of a transition metal dichalcogenide (TMDCs) such as MoS_2_ and graphene, forming an heterostructure is very interesting, since it combines the excellent optical properties of MoS_2_ and the high mobility and transparency of graphene^9^,^10^,^11^,^12^.

One well-established method to produce high quality wafer-scale monolayer graphene is the epitaxial approach based on the graphitization of the Si face of SiC^13^,^14^. A considerable advantage of this technique lies in the fact that the wide band gap semiconductor SiC wafers can be employed as a substrate, so that no additional transfer step is required to conduct electrical or optical measurements. To our knowledge, until now there are few works discussing the possibility of growing TMDCs materials (MoS_2_ or WS_2_) on epitaxial graphene/SiC^15^,^16^,^17^,^18^. In particular a direct growth approach by Chemical Vapor Deposition (CVD)^15^,^16^,^18^,^19^,^20^,^21^ or metal-organic chemical vapour deposition (MOCVD)^17^ is generally used. Even if the authors obtained high quality interfaces between TMDCs film and graphene, the direct growth method suffers of the fact that the obtained TMDC grain size is small (ranging from hundreds of nm to few microns). However the possibility to obtain large area TMDCs/graphene heterostructures is important for a large variety of *in situ* characterization techniques, and is also a basic requirement for realistic applications. Recently Han *et al*. developed a novel seeded CVD patterned growth method to obtain highly crystalline MoS_2_ flakes on oxidized silicon substrate^22^. MoS_2_ grown by this approach has high crystallinity, with large flakes (between 20–100 μm) and electrical and optical properties comparable to exfoliated material. These CVD-grown flakes are suitable for transfer onto epitaxial graphene/SiC substrate in order to obtain large area of MoS_2_/graphene heterostructures. Using a transfer process (see results and discussion and Fig. 1(a)) we have obtained monolayer and bilayer MoS_2_/graphene heterostructures, allowing the study of interlayer interaction between TDMCs materials and graphene on a large scale, using several complementary techniques. Angle resolved photoemission spectroscopy (ARPES) measurements were used to study the electronic structure of the MoS_2_/epitaxial graphene heterostructure. Due to a weak interlayer coupling the electronic structure of graphene and MoS_2_ are well retained in their respective layers. However the band alignment in the MoS_2_/graphene heterostructure allows under illumination a charge transfer process from MoS_2_ to graphene. This is revealed by a downshift of the graphene 2D Raman bands, an upshift of the A_1g_ Raman band of MoS_2_, and a strong quenching of photoluminescence (PL) of MoS_2_/graphene heterostructure. Complementary we performed photocurrent measurements to elucidate how the presence of the semiconductor affects the photoconductive properties of graphene. In that respect, this work may open a new way in graphene optoelectronics by modulating the graphene photoelectric response through 2D materials interfacing.

## Results and Discussions

**Figure 1:**
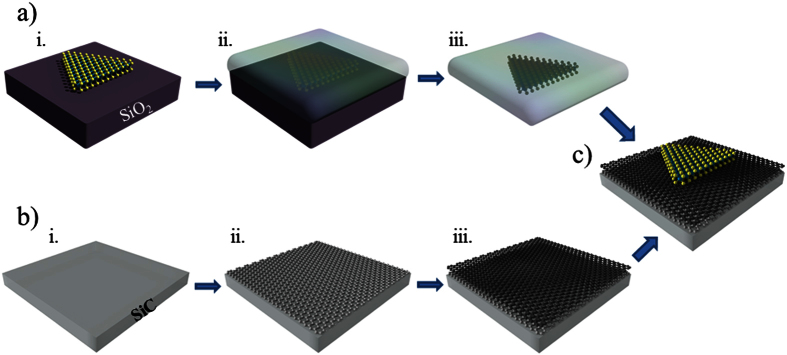
Schematic transfer process of MoS_2_ on epitaxial grapheme: (**a**) i. Growth of MoS_2_ on Si/SiO_2_. ii. Coating of PMMA on the top of the sample iii. KOH etching of SiO_2_ substrate. (**b**) i. 4H**-**SiC substrate. ii. Interface layer. iii. Monolayer epitaxial Graphene. (**c**) Monolayer MoS_2_ on epitaxial grapheme.

**Figure 2:**
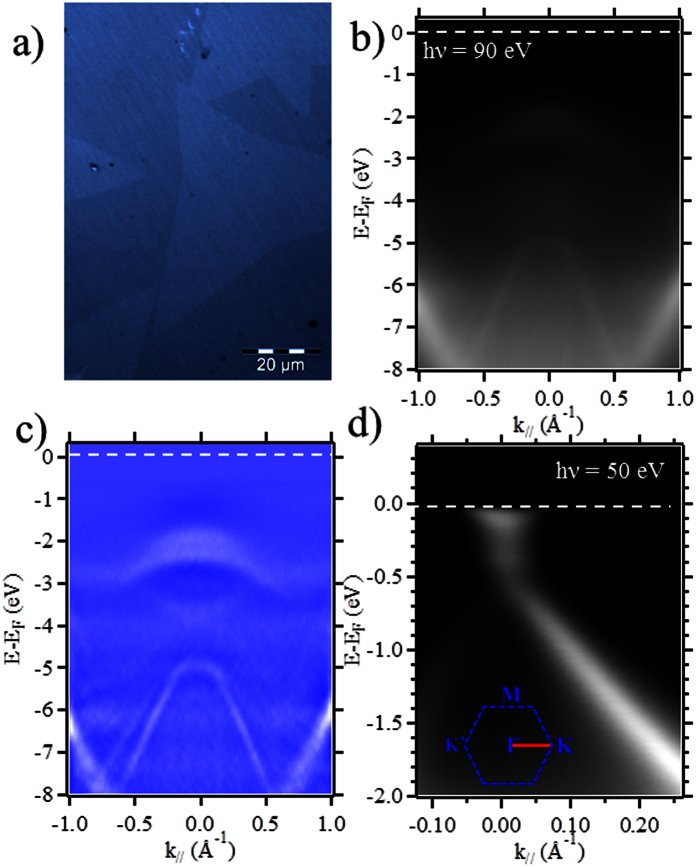
ARPES measurement for monolayer and bilayer MoS_2_/graphene heterostructures: (**a**) typical optical image of the MoS_2_ transferred on epitaxial graphene layer, (**b**) ARPES measurements of MoS_2_/graphene measured at hν = 90 eV along to the K′ΓK direction of graphene Brillouin zone, and (**c**) Second-derivative spectra of (**b**) to enhance the visibility of the bands. (**d**) ARPES spectrum around the K point of graphene of the MoS_2_/graphene heterostructure at hv = 50 eV. The white dotted lines in panels (**b**–**d**) indicate the Fermi level position.

TMDCs/graphene heterostructures were made from MoS_2_ flakes grown by chemical vapor deposition on oxidized silicon substrates that were then transferred onto epitaxial graphene grown on SiC(0001) (Fig. 1(a)). The graphene underlayer used in this study was obtained by annealing 4H-SiC(0001) (see methods) (Fig. 1(b)i–iii). The CVD growth procedure of MoS_2_ on SiO_2_ results in characteristic single-crystal domains shaped as well-defined equilateral triangles^22^ (Figure S1). The single- crystal flakes with mono, bi and multilayer thicknesses were identified by their optical contrast and characteristic triangular shape, and further confirmed by micro-Raman, and micro photoluminescence (micro-PL) measurements. For the transfer step, we spin-coated PMMA onto the MoS_2_ flakes and peeled them off from the SiO_2_ substrate by wet etching in KOH solution (Fig. 1(a)ii,iii). Afterward, we transferred the PMMA/MoS_2_ layer onto the graphene/SiC substrate (Fig. 1(c)). We finally removed the PMMA using acetone. Due to the high density of MoS_2_ flakes on the Si/SiO_2_ substrate (50% of the total area of the sample), we were able to obtain several flakes with various stacking order and orientation in a single transfer step (Fig. 2(a)). The MoS_2_ domains transferred onto the graphene retain their triangular shapes with lateral sizes of ~20 to ~100 μm. To further clean the surface and interface of the MoS_2_/graphene heterostructure, we annealed the samples at T = 300 °C for 30 mn in UHV (base pressure below P ~ 10^−10^ mbar). For the following experiments, the monolayer, bilayer and multilayer coverage was estimated from optical analysis to be around 83%, 15% and 2% respectively.

**Figure 3:**
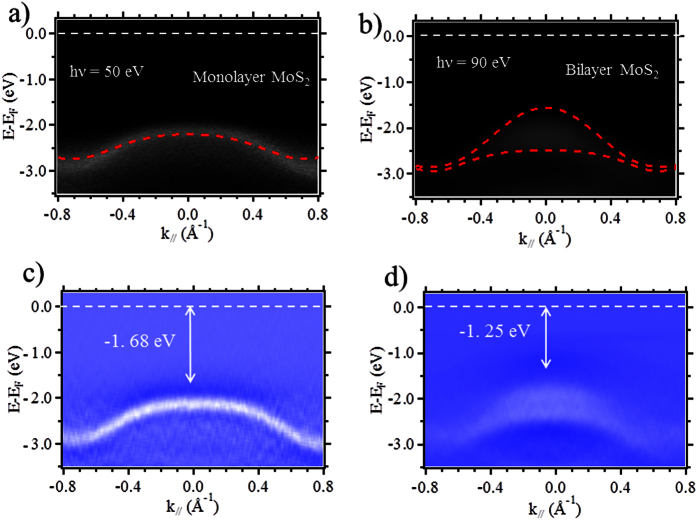
Electronic structure of monolayer and bilayer MoS_2_/graphene heterostructures: (**a,b**) ARPES spectra and calculated band structures (red dotted lines) of monolayer and bilayer MoS_2_ thin films at hν = 50 and 90 eV respectively. (**c,d**) Second-derivative spectra of (**a,b**), respectively, to enhance the visibility of the bands. White dotted lines indicate the Fermi Level position. The arrows indicate the distance of the top of the valence band at the Γ point to the Fermi level.

In order to better understand the electronic properties of the MoS_2_/graphene/SiC heterostructure, we measured its band structure by angle-resolved photoemission spectroscopy (ARPES) at the Cassiopée beamline of Synchrotron Soleil. The small x-ray spot size (50 × 50 μm^2^) allowed the measurement of the band structure of a single flake (monolayer or bilayer) forming the MoS_2_/graphene heterostructure. The photoelectron intensity is presented in Fig. 2(b) as a function of energy and k-momentum, along the K′–Γ–K direction of the first graphene Brillouin zone. The second-derivative spectrum in Fig. 2(c) is provided to enhance the visibility of the band structure. The zero of the binding energy (i.e., the Fermi level) was determined by fitting the leading edge of the graphene layer at the same photon energies and under the same experimental conditions. Beside the typical linear dispersion of the π bands of graphene, a new set of bands is visible at the Γ point of the Brillouin zone independently of the orientation angle between the flake and the graphene underlayer, which is the signature of MoS_2_ valence band. A close inspection of the K point of graphene Brillouin zone is shown in Fig. 2(d). This spectrum is obtained orienting the sample along the Γ-K direction of the graphene Brillouin zone. In this case the mismatch angle between MoS_2_ flake and graphene underlayer is critical. In order to obtain a perfect alignment of the Brillouin zone of the two materials we need a mismatch angle of zero degree. As we can see in Fig. 2(d), along the Γ-K direction, we only see the graphene signature. The two spin split bands expected at the K(K′) point^23^,^24^ of MoS_2_ are not visible indicating a non-zero mismatch angle for this flake. However we clearly see the graphene band structure, in particular the π bands of graphene preserve their linearity characteristic of a massless Dirac fermions signature, indicating a high structural quality of the MoS_2_/graphene heterostructure. Moreover, similar to pristine monolayer graphene, the Dirac point (E_D_) is located at 0.3 eV below the Fermi level (FL). From a linear fit, using the relation E = *ħv*_*F*_*k*, we obtain the value of the Fermi velocity *v*_*F*_ ~ 1.1 × 10^6^ m/s, which matches the expected value for monolayer graphene on SiC. As the linear dispersion and Fermi velocity of the pristine graphene is preserved in the MoS_2_/graphene heterostructure, we can infer that the MoS_2_ transfer does not affect the electronic structure of monolayer graphene in the heterostructure formation. In addition, this feature is expected theoretically since in van der Waals heterostructures, the superposition of each layer electronic structure constitutes a good approximation of the electronic structure of the multilayer. Differently from previous work of Diaz *et al*. no signature of interlayer hybridization^25^ is present on the π-band of graphene. This is probably due to the mismatch angle between the MoS_2_ flake and the graphene underlayer. However identifying the dependence of this effect on the mismatch angle would clearly require a further work and go much beyond the main objective of this paper. Figure 3(a,b) show a direct comparison of the calculated band structures (see method) and the corresponding ARPES spectra of the monolayer and bilayer MoS_2_ on epitaxial graphene, along the K-Γ-K direction in the hexagonal Brillouin zone, the respective second derivative are shown in Fig. 3(c,d). Comparisons with our Density Functional Theory (DFT) calculations clearly show that the monolayer, bilayer-dependent band structure evolution shows excellent agreement with theoretical calculations. Monolayer MoS_2_ presents only one band at the Γ point (maximum at Binding Energy (BE) ~ −1.68 eV ± 0.05 eV), and this structure evolves into two branches in the case of bilayer MoS_2_ (maximum at BE ~ −1.25 eV ± 0.05 eV). This evolution is representative of the splitting of the bands due to the weak van der Waals interaction between the two MoS_2_ layers. The relative position of the top of the valence bands at the Γ point in the bilayer film is closer to the Fermi level than the one obtained from the monolayer (Figure S2). This indicates that MoS_2_ undergoes a crossover from an indirect to a direct bandgap in monolayer^9^,^23^,^26^, as predicted theoretically (Figure S3). Indeed, we can observe from Figure S3 (a) to (c) the evolution of the band structure of MoS_2_ from monolayer to bi- and trilayer, calculated in DFT. Even though the trilayer MoS_2_ has not been considered in details here experimentally, we show the corresponding DFT result to exhibit the evolution of the band structure when considering multilayer MoS_2_. In the bi- and trilayer systems, the top of the valence band is located at the Γ point, yielding an indirect band gap with the bottom of the conduction band between the K (K′) and Γ points. However, when considering the monolayer band structure, the top of the valence band becomes very flat near the Γ point, leading to a direct gap at the K (K′) point. The evolution of the valence band at the Γ point provides a straightforward method to identify the thickness of ultrathin MoS_2_ films, and also proves the high quality of MoS_2_ transferred on epitaxial graphene. The epitaxial graphene underlayer does not affect the MoS_2_ band structure, as expected for a van der Waals heterostructure. However, we do not exclude the presence of the universal buckled form of 2D crystal in our MoS_2_ layer^3^.

To further investigate the electronic properties of mono and bilayer MoS_2_/graphene heterostructure, micro photoluminescence (micro-PL) and micro-Raman measurements are performed at room temperature (see method).

**Figure 4:**
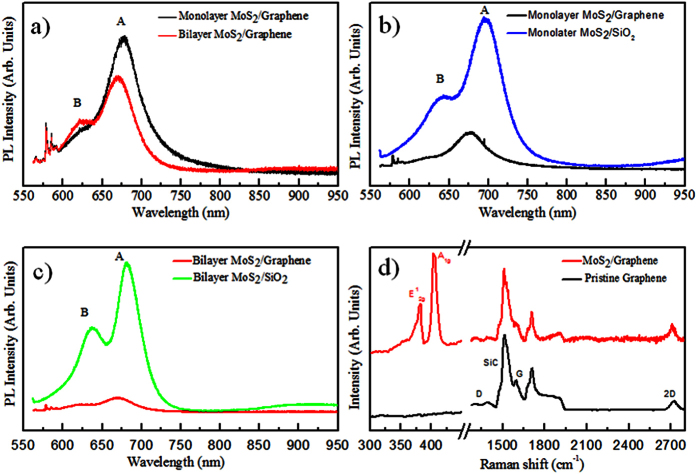
Photoluminescence and Raman spectra for monolayer and bilayer MoS_2_/graphene heterostructures: (**a**) Photoluminescence spectra of MoS_2_/graphene heterostructures sample for monolayer (black data) and bilayer (red data) MoS_2_, (**b,c**) Photoluminescence spectra of MoS_2_/graphene heterostructures and MoS_2_/SiO_2_ sample for monolayer (MoS_2_/graphene black data and MoS_2_/SiO_2_ blue data) and bilayer respectively (MoS_2_/graphene red data and MoS_2_/SiO_2_ green data), (**d**) Graphene wavenumber region of the Raman spectra of pristine graphene (black data) and graphene capped with MoS_2_ (red data).

The PL spectra present the two characteristic excitonic peaks A and B^27^,^28^,^29^,^30^,^31^ originating from the transition at the K-point of the Brillouin zone (Fig. 4(a)). The spectra also reflect the band structure change from indirect band gap semiconductor of 2ML MoS_2_ to direct gap semiconductor in monolayer MoS_2_. The PL signal is strongly enhanced when a direct band gap is present. The same behavior was also observed for the as grown MoS_2_ on SiO_2_ (Figure S4). From the PL spectrum we can extrapolate the band gap value, which corresponds to 1.83 eV in agreement with our DFT calculations and previous experimental works^32^,^33^,^34^,^35^,^36^.

In the case of vertical vdW heterostructures, possible PL intensity variations could arise from the interference effects due to different optical constants and thickness of the different layers forming the heterostructure^37^. Buscema *et al*. defined in their work^37^ a substrate-dependent enhancement factor Γ^−1^ which allows a normalization procedure of the spectra taking into account the effect of optical interferences^37^, for the ML MoS_2_ on different substrate. Following their results we can see that Γ^−1^ ~ 1 in the case of SiO_2_ substrate. In the case of very thick FLG (15 nm), Γ^−1^ is between 8–14, and presents a huge decrease as the thickness of graphene layer is reduced (~2 in the case of 5 nm FLG). In our case, we have only one graphene layer, meaning that as for SiO_2_ we can suppose Γ^−1^ ~ 1. Therefore, in both cases we can neglect the effect of optical interferences, and directly compare the PL raw data of MoS_2_/SiO_2_ and MoS_2_/graphene heterostructures as shown in Fig. 4(b,c). In MoS_2_/SiO_2_ heterostructure strain can exist due to the different thermal expansion coefficients of MoS_2_ and the SiO_2_ substrate during the MoS_2_ flakes growing^38^. The transfer process on the graphene substrate due to the weak van der Waal forces at the interface releases the lattice strain^39^. This effect is reflected on the PL spectrum as a blue-shift of the A peak in the MoS_2_/graphene heterostructure^38^,^39^,^40^. Moreover, the PL signal in the case of graphene underlayer is strongly quenched (about 60–70% with respect to the SiO_2_ substrate). This phenomenon was explored also as a function of the laser power. In Figure S5 the integrated PL intensity as a function of excitation power is shown. As expected in this range of powers (between 0.5 mW and 25 mW) the MoS_2_ PL intensity evolves linearly with increasing laser excitation^41^,^42^ for both substrate SiO_2_ and graphene. But in the case of graphene underlayer the PL signal is quenched for each laser power.

This phenomenon is the signature of electron transfer from MoS_2_ to the graphene which hinders the recombination of electron−hole pairs created by the photoexcitation^32^,^43^. This electron transfer is not attributed to a strong coupling between MoS_2_ and graphene (since we consider a weak van der Waals interaction for this structure), but rather to a standard hopping from an electron in MoS_2_ conduction band to an unoccupied state at the same energy in graphene^43^,^44^.

**Figure 5:**
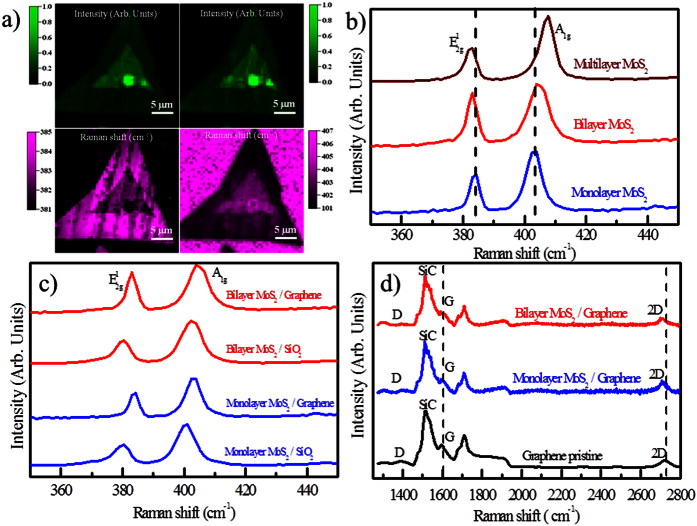
Raman results for monolayer and bilayer MoS_2_/graphene heterostructures: (**a**) Raman maps images of the peaks position and the peaks intensity of the 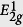 and A_1g_ modes of MoS_2_ on epitaxial graphene, (**b**) Raman spectra of monolayer, bilayer and multilayer MoS_2_ on graphene, (**c**) Comparison of the Raman spectra of monolayer and bilayer MoS_2_ on epitaxial graphene and on SiO_2_. (**d**) Raman spectra of pristine graphene and MoS_2_/graphene heterostructures sample.

Figure 4(d) shows typical Raman spectra of the MoS_2_/graphene heterostructure and pristine graphene layer^45^ in the wavenumber range of 300–2800 cm^−1^. Besides the typical second-order Raman bands that originate from the SiC substrate, the three main structures typical of graphene are present on the pristine graphene and MoS_2_/graphene spectra: i) the D band (defect induced mode), ii) the G band (in-plane vibration mode) and iii) the 2D band (two-phonon mode)^46^. In the case of MoS_2_/graphene heterostructure within the wavenumber range between 350–450 cm^−1^ two new peaks are present. These two characteristic features correspond to the in-plane vibration (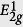) and out of plane (A_1g_) of Mo and S atoms in the MoS_2_ film^34^,^47^. The intensity and Raman shift (Δ) maps of the A_1g_ and 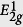 are shown in Fig. 5(a). The intensity and the Raman shift Δ increase with the number of MoS_2_ layers. The average Raman spectra obtained from each layer are shown in Fig. 5(b). The obtained values of Δ are ~19 cm^−1^, ~21 cm^−1^ and 24 cm^−1^ corresponding to monolayer, bilayer and multilayer (~three layers) MoS_2_, respectively^34^,^47^. To further examine the role of the interaction with the substrate, we compare the MoS_2_ on graphene and on SiO_2_ substrates (Fig. 5(c)). After the transfer we observed that the A_1g_ and 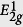 of the 1ML and 2ML MoS_2_ on graphene upshifted by about 4 and 3 cm^−1^ and 3 and 2 cm^−1^, respectively, meanwhile the line widths of these peaks, calculated by the full widths at half maximum (*fwhm*) result of a Lorentzian fitting, are narrower than those on SiO_2_ (~3 cm^−1^ smaller for 1 ML and ~2 cm^−1^ for the 2 ML for both peaks). As in the case of the PL spectra, the upshift of the in plane Raman mode (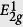) is the result of tensile strain release after the MoS_2_ transfer on the graphene underlayer^38^,^39^,^40^,^48^. The A_1g_ mode shows a weaker strain dependence than the 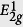. Consequently, its large upshift upon the transfer can be explained as the result of additional effects: i) the establishment of a van der Waals interaction between MoS_2_ and graphene^47^, and ii) a decrease in the electron concentration of the MoS_2_^49^. This latest, as shown by the micro-PL measurements is due to a charge transfer under illumination from MoS_2_ to graphene. As illustrated by Zhou *et al*., the important upshift of the A_1g_ is also a signature of the high quality of interface between MoS_2_ and graphene^48^. Moreover, when MoS_2_ is transferred from SiO_2_ to graphene the reduced substrate surface roughness and impurities as well as the similar lattice structure are responsible for the narrowing of the MoS_2_ Raman features^50^.

A detailed analysis of the Raman spectra of the monolayer and bilayer MoS_2_/graphene heterostructure and pristine graphene layer in the wavenumber range of 1300–2800 cm^−1^ are shown in Fig. 5(d). As explained before the presence of graphene is indicated by three main structures: i) the D band, ii) the G band and iii) the 2D band. The D peak is small (~1% of the G peak intensity), indicating the high quality of pristine graphene. The intensity of this peak did not increase after transfer, suggesting that the MoS_2_ transfer process did not induce defects in the graphene substrate. The Raman spectra of the graphene below MoS_2_ domains showed clear differences from that of the free-graphene areas. First, we observe a broad background which increases with higher wavenumbers. This background comes from the PL of MoS_2_, which confirms the presence of both graphene and MoS_2_ in the measured area. Second, the intensity of the 2D band was reduced by MoS_2_. Third, both the G and the 2D bands are shifted. In pristine graphene the 2D band is located at 2722 cm^−1^ and it is shifted at 2710, and 2708 cm^−1^ for monolayer and bilayer MoS_2_, respectively. There are two factors that can influence the Raman 2D band position: charge transfer^45^,^51^,^52^, and strain^53^,^54^,^55^. In our Raman measurements, the spectra were taken at room temperature and the laser power was low (~5 mW) to avoid the influence of laser heating. Thus, the observed 2D band downshift does not originate in differences of temperature, which can induce different strain in graphene and MoS_2_. It is known that depending on the introduced carriers, the 2D band position shifts differently^56^, with up- and downshifts corresponding to hole and electron doping, respectively^51^. Then in MoS_2_/graphene heterostructure this downshift indicates an increase in the electron concentration in graphene under illumination (n-type doping)^53^. This phenomenon is in agreement with the up-shift of the Raman feature of the MoS_2_, and confirms that the photoelectrons generated by the Raman laser are transferred from the MoS_2_ to graphene. At the same time the G peak presents an upshift. If we focus our attention on monolayer MoS_2_/graphene we have an upshift of ~3 cm^−1^ ± 1cm^−1^. From this shift we can obtain a quantitative analysis of the level of electron doping under illumination^53^,^57^,^58^,^59^. In fact the G peak frequency blue-shifts linearly with the Fermi level position as 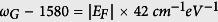. From this expression, we estimate the Fermi level position with respect to the Dirac point for pristine graphene and 1ML MoS_2_/graphene as 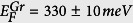 and 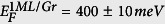, respectively. Then the upshift of the G mode of ~3 cm^−1^ implies a change in the graphene Fermi level of ~70 meV. From these values we can calculate the variation in the n-doping of graphene using the relation 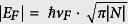, and the value of the Fermi velocity obtained before by the linear fit of the Dirac cone in Fig. 2(d) (*v*_*F*_ = 1.1 × 10^6^ m/s), we obtained an electron density for pristine graphene |*N*| ~ 7 × 10^12^ cm^−2^ which increases to |*N*| ~ 10^13^ cm^−2^ for 1ML MoS_2_/graphene under illumination.

**Figure 6:**
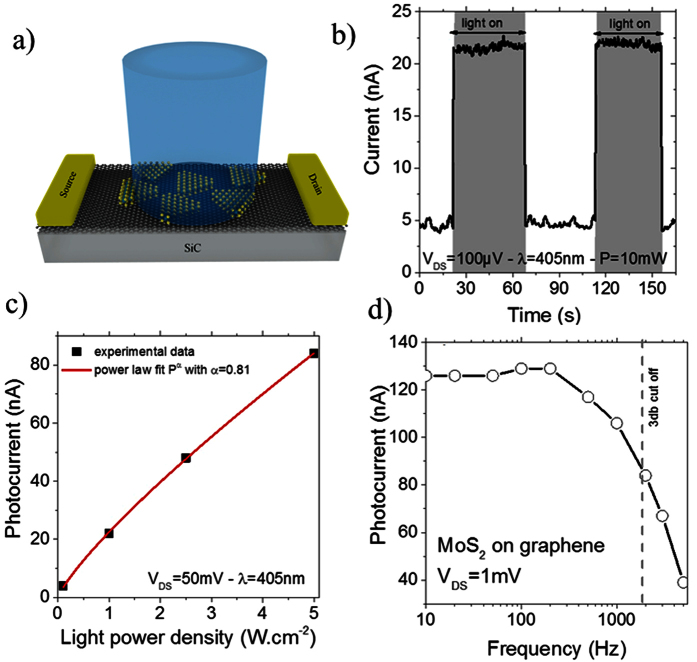
(**a**) Scheme of the device based on epitaxial graphene MoS_2_ flakes, (**b**) Current as a function of time under constant applied electric field while the light is turned on and off (V_DS_ = 0.1 mV − λ = 405 nm), (**c**) photocurrent as a function of the light intensity under constant applied electric field (V_DS_ = 50 mV − λ = 405 nm), (**d**) frequency dependence of the photocurrent under constant applied electric field and light intensity.

Phototransport properties of the sample are investigated in a planar geometry (i.e. the electrodes are connected to MoS_2_ decorated graphene) at room temperature while illuminating the sample with light energy (λ = 405 nm (hν ≈ 3 eV)) above the MoS_2_ band gap (Fig. 6(a)). We prepared a lateral device by standard optical lithography. We used dry etching to define a graphene mesa and titanium/gold contacts (20/200 nm). Compared to pristine graphene the photoresponse of the vdW heterostructure is significantly enhanced and a clear modulation of the current is observed under illumination, see Fig. 6(b). The generated photocurrent presents almost no dependence with the applied bias (Figure S6). Similar behavior has already been observed and attributed to thermoelectric effect^60^ and the later result from the inhomogeneous distribution of the MoS_2_ flake at the scale of the light source spot. On the other hand, if the size of the device is reduced down to a single MoS_2_ flake, the phototransport is very different since we observe a more usual photoconductive behavior, where the slope of the I-V curve chnage under illumination (Figure S7).

Since the spot diameter of the laser is 1 mm^2^, and the density of the MoS_2_ fakes is about 30% of the sample we can estimate the responsivity to be around 6 μA.W^−1^. This limited value is the result of the limited absorption of the semiconductor (MoS_2_) because of its thickness and limited coverage. The photocurrent generation in TMDC heterostructure generally suffers from two main limitations from an applied point of view, which are their slow response time^35^ and their strong dependence of the photoresponse with the light intensity^61^,^62^. In the following we investigate these two properties. We measured the light intensity dependence of the response and found an almost linear dependence of the current with the photon flux (Φ) see Fig. 6(c). More precisely the power dependence of the current (I) can be fitted using a power low^44^, IαΦ^0.8^. Such dependence is weaker than for large gain system like graphene–PbS quantum dot hybrid system^63^, which allows using MoS_2_ decorated graphene system over a larger range of light flux. While modulating the incident laser intensity thanks to a signal generator we can extract the frequency dependence of the graphene-MoS_2_ system^64^. The 3dB cut off frequency is measured to be 2 kHz, see Fig. 6(d).

In summary, we have studied the electronic properties of the wet transfer of large area MoS_2_ on epitaxial graphene layer. From the PL, ARPES and micro-Raman data presented in Figs 1, 2, 3, it is clear that the MoS_2_/graphene heterostructure presents a good long-range order at large scale. Our ARPES measurements on the heterostructure showed that graphene and MoS_2_ largely retained their original electronic structure indicating weak van der Waals interactions between the two crystals. The PL quenching in the MoS_2_-graphene heterostructure and the upshift of the A_1g_ Raman mode of MoS_2_ and the downshift of the 2D Raman mode of graphene confirm the charge transfer between the MoS_2_ and graphene layer. Our work suggests that the optical properties of MoS_2_ are strongly affected by the underlayer graphene. The fact that an interaction is visible between MoS_2_ and graphene is a clear signature of the quality of the wet transfer process and of the absence of interfacial contaminations. Moreover, this charge transfer can be influenced by the underlayer graphene doping, varying the band alignment in the heterostructure, which should be considered in device design and fabrication. Furthermore, efficient photoresponse was observed on large 2D MoS_2_/epitaxial graphene devices, opening a new way in graphene optoelectronics.

## Methods

### Growth of graphene/SiC(0001)

Monolayer graphene studied in this paper is produced via a two-step process beginning with a starting substrate of 4H-SiC(0001)^65^. Prior to graphitization, the substrate is hydrogen etched (100% H_2_) at 1550 °C to produce well-ordered atomic terraces of SiC. Subsequently, the SiC sample is heated to 1000 °C at a pressure of about 10^−5^ mbar and then further heated to 1550 °C in an Ar atmosphere. This graphitization process results in the growth of an electrically active graphene layer on top of the buffer layer, covalently bound to the substrate^66^. The sample was cooled down to room temperature and transferred *ex-situ* to perform different measurements.

### Growth of MoS_2_/SiO_2_/Si(001)

MoS_2_/SiO_2_ samples were grown via CVD in a 1″ quartz tube furnace. Microliter droplets of saturated ammonia heptamolybdate solution were dried onto the corners of a Si/SiO_2_ growth substrate that had previously been coated with a layer of sodium cholate (1% solution spin coated 4000 rpm for 60 sec). Sodium cholate is a known growth promoter, acting to increase diffusion of the molybdenum source by increasing the surface adhesive energy relative to the adatom cohesive energy^22^. The growth substrate was placed in the center of the furnace and heated to 800 °C. A 25 mg sulfur pellet was placed on a piece of silicon and positioned upstream in the furnace such that its temperature was approximately 150 °C. Carrier gas (500 sccm N_2_) was used to bring sulfur vapor into the furnace for a 30 min growth period. The sample was then rapidly cooled by cracking open the furnace and sliding it downstream with respect to the quartz tube.

### Characterization of MoS_2_/graphene heterostructure

The PL measurements were carried out using a confocal commercial Renishaw micro-Raman microscope with a 100× objective and a Si detector (detection range up to ~2.2 eV). The Raman spectra measurements were performed on the same microscope using a 532 nm laser in an ambient environment at room temperature. To ensure the reproducibility of the data, we followed a careful alignment and optimization protocol. In addition, the excitation laser was focused onto the samples with spot diameter of ~1 μm and incident power of ~5 mW. The integration time was optimized to obtain a satisfactory signal-to-noise ratio. We obtained Raman spatial maps by raster scanning with 0.3 μm step size using a precision 2D mapping stage.

The ARPES measurements were conducted at the CASSIOPEE beamline of Synchrotron SOLEIL (Saint-Aubin, France). We used linearly polarized photons of 50 eV and 90 eV and a hemispherical electron analyzer with vertical slits to allow band mapping. The total angle and energy resolutions were 0.25*°* and 16 meV. The mean diameter of the incident photon beam was smaller than 50 *μ*m. All ARPES experiments were done at room temperature.

For electrical measurements the samples are electrically characterized in air at room temperature. Temporal and power dependence of the current are obtained while biasing the sample and measuring the current with a Keithley 2634B as sourcemeter. Illumination is ensured by a 405 nm blue laser diode with tunable light intensity. The frequency dependence of the photocurrent is measured while the sample is biased using a Keithley 2634B. The output signal is amplified in a Keithley 427 current amplifier and the signal acquired on HP oscilloscope.

### DFT calculations

First-principles calculations have been performed using a very efficient DFT localized orbital molecular dynamic technique (FIREBALL)^67^,^68^,^69^,^70^. Basis sets of sp^3^d^5^ for S and Mo were used with cutoff radii (in atomic units) s = 4.3, p = 4.7, d = 5.5 (S) and s = 5.0, p = 5.6, d = 4.8 (Mo). In this study we have considered standard unit cells of 3, 6 and 9 atoms to describe respectively a mono-bi- and trilayer of MoS_2_. Each configuration has been relaxed using a sample of 32 k-points in the Brillouin zone. In case of multilayer MoS_2_, we have considered the most stable AB stacking, and the equilibrium distance has been determined using the LCAO-S^2^ + vdW formalism^71^,^72^. Finally, a set of 300 special k points along the K′–Γ–K path has been used for the band structure calculations. The corresponding band structures as well as extended atomic representations of the multilayers MoS_2_ are provided in Fig. S3(a–f) in the Supplementary Informations.

## Additional Information

**How to cite this article**: Pierucci, D. *et al*. Large area molybdenum disulphide - epitaxial graphene vertical Van der Waals heterostructures. *Sci. Rep.* **6**, 26656; doi: 10.1038/srep26656 (2016).

## Supplementary Material

Supplementary Information
