# Supplementary material for: Large area molybdenum disulphide- epitaxial graphene vertical Van der Waals heterostructures
Source: Sci Rep. 2016 Jun 1;6:26656. doi: 10.1038/srep26656 (PMC4894673; doi:10.1038/srep26656)
Supplement: Supplementary Information [file srep26656-s1.pdf]

Supplementary information

Large area molybdenum disulphide - epitaxial graphene vertical Van der Waals heterostructures

Debora Pierucci[1,†], Hugo Henck[1, †], Carl H. Naylor[2], Haikel Sediri[1], Emmanuel, Lhuillier[3], Adrian Balan[4], Julien E. Rault[5], Yannick J. Dappe[6], François Bertran[5], Patrick Le Fevre[5], A.T Charlie Johnson[2] and Abdelkarim Ouerghi[1*]

[1] Laboratoire de Photonique et de Nanostructures (CNRS- LPN), Route de Nozay, 91460 Marcoussis, France
[2]Department of Physics and Astronomy, University of Pennsylvania, 209S 33rd Street, Philadelphia, Pennsylvania 19104, USA
[3] Institut des Nanosciences de Paris, UPMC, 4 place Jussieu, boîte courrier 840, 75252 Paris cedex 05, France
[4]Laboratoire d'Innovation en Chimie des Surfaces et Nanosciences, DSM/NIMBE/LICSEN (CNRS UMR 3685), CEA Saclay, 91191 Gif-sur-Yvette Cedex, France
[5] Synchrotron-SOLEIL, Saint-Aubin, BP48, F91192 Gif sur Yvette Cedex, France
[6]Service de Physique de l'Etat Condensé, DSM/IRAMIS/SPEC (CNRS UMR 3680), CEA Saclay, 91191 Gif-sur-Yvette Cedex, France

*Corresponding author, E-mail: abdelkarim.ouerghi@lpn.cnrs.fr

[†]These authors contributed equally to this work

This Supporting Information includes: Figures S1 to S6.

S1. CVD growth of MoS_2 on SiO_2

Large scale MoS_2 monolayer flakes (~20 to ~100 μm) were obtained by CVD on oxidized silicon substrate. The monolayer MoS_2 flakes were then easily identified by their optical contrast with respect to the substrate (Figure S1) and confirmed by micro-Raman spectroscopy.

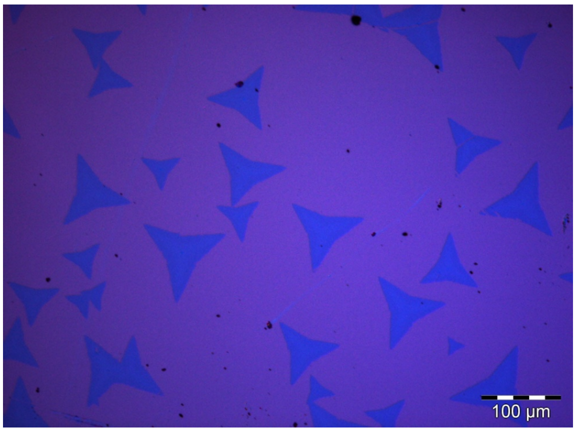

Figure S1: Optical image of CVD grown MoS_2 flakes on SiO_2.

S2. Determination of the local Valance band Maximum (VBM) from ARPES measurements

From the intersection of the linear extrapolation of the leading edge of the valence band spectrum with the base line, we could locate the position of the valence band maximum (VBM) for the MoS_2 in the heterostructure, for mnolayer and bilayer MoS_2.

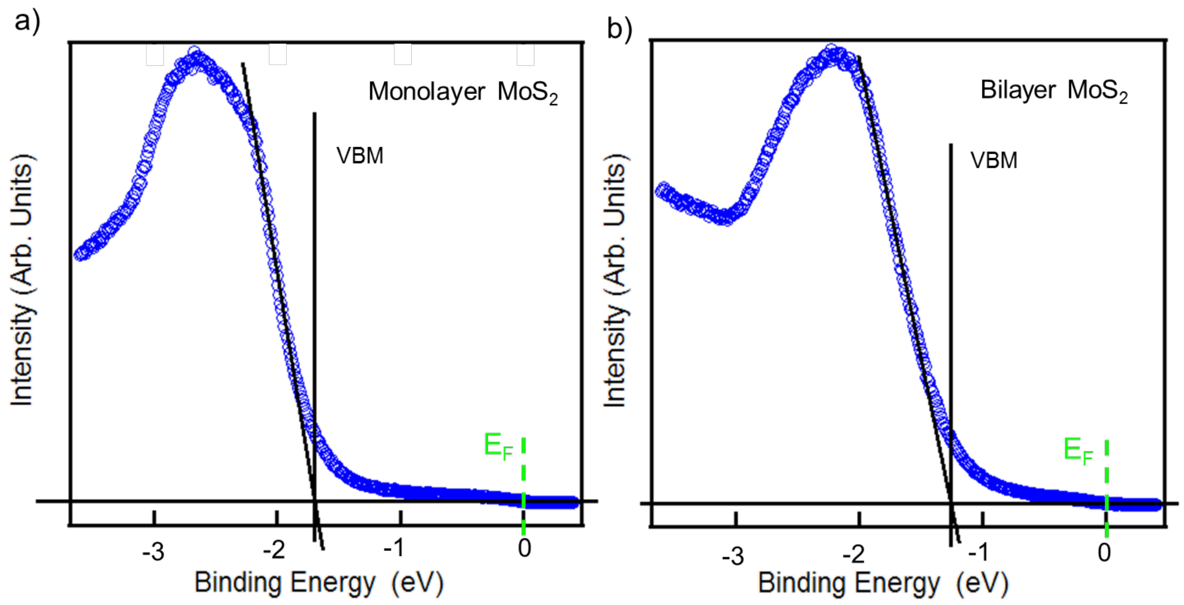

Figure S2: Integrated valence band (VB) around the Γ point of MoS_2 Brillouin zone: a) Monolayer MoS_2 on epitaxial graphene/SiC(0001), b) Bilayer MoS_2 on epitaxial graphene/SiC(0001). The local valence band maximum (VBM) at the Γ point is obtained by the intersection of the linear extrapolation of the leading edge of the VB spectrum with the base line (black lines).

S3. DFT calculations

Our DFT calculated band structures along the K- Γ-K' of monolayer, bilayer and trilayer MoS_2 (Figure S3) shows that the MoS_2 presents a direct to indirect bandgap transition for the 2 ML thickness.

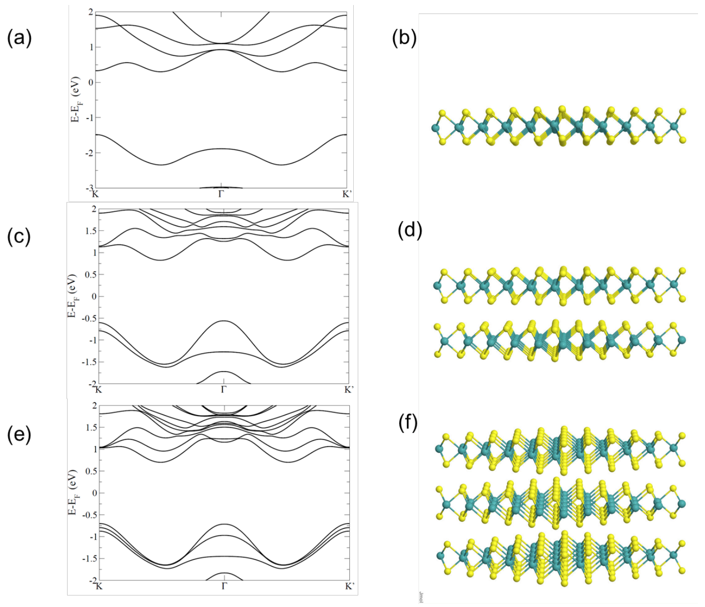

Figure S3: DFT calculated band structures along the K- Γ-K' path of MoS_2 a) monolayer, c) bilayer and e) trilayer. Corresponding extended atomic structures are represented in b), d) and f).

S4. PL spectra of monolayer and bilayer MoS_2 on SiO_2

The PL signal is strongly enhanced when a direct band gap is present (figure 4). The PL spectra present the two characteristic excitonic peaks originating from the transition at the K-point of the Brillouin zone. From the PL spectrum we can extrapolate the band gap value, which corresponds to 1.83 eV in agreement with our DFT calculations.

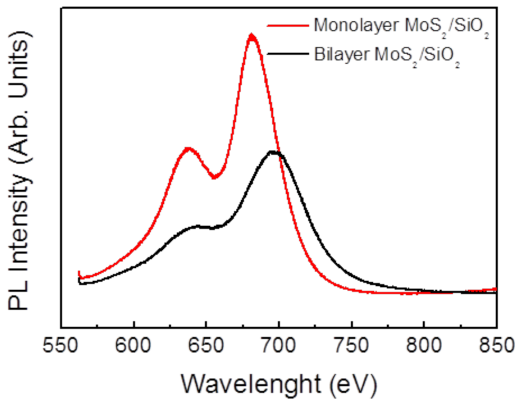

Figure S4: Photoluminescence spectra for monolayer (red curve) and bilayer (black curve) MoS_2 on SiO_2

S5. PL measurements as a function of laser power: MoS_2 on SiO_2 and MoS_2 on graphene

PL quenching was studied as a function of the laser power. We compared the PL signal for MoS_2 on SiO_2 and MoS_2 on graphene for different values of laser power: 0.5 mW, 2.5 mW, 5 mW and 25 mW. For each spectrum we used the smaller acquisition time allowed for our experimental set-up (10 s) in order to avoid laser-induced thermal effects. In the case of MoS_2 on SiO_2 for 25 mW we had a saturation of the detected PL signal. In the figure below we represent the integrated PL intensity as a function of the laser power. As expected the PL signal is proportional to the laser power (Kom *et al.* Appl. Phys. Lett. 99, 102109 (2011) and Ko *et al.* Journal of Nanoscicnece and Nonotechnology vol 15, 1-4, 2015) for both substrates. The quenching of the PL signal in the case of the graphene substrate is evident for each laser power.

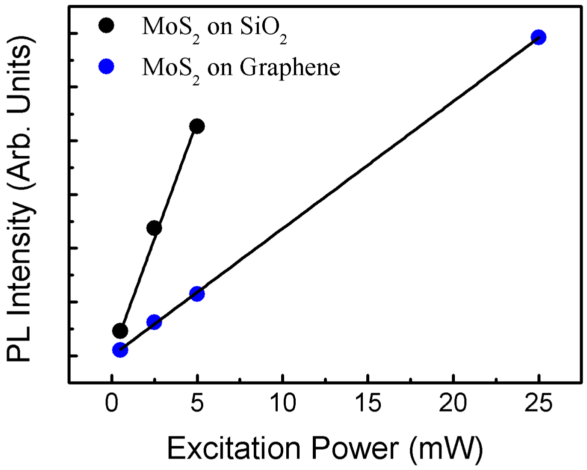

Figure S5: Integrated PL intensity for different excitation powers: 0.5 mW, 2.5 mW, 5 mW and 25 mW for MoS_2 on graphene (blue dots) and MoS_2 on SiO_2 (black dots). For each spectrum we used the smaller acquisition time allowed for our experimental set-up (10 s) in order to avoid laser-induced thermal effects. In the case of MoS_2 on SiO_2 for 25 mW we had a saturation of the detected PL signal. The black lines represent a linear fit of data.

S6. Photocurrent measurements for MoS_2 on graphene

Concerning the bias dependence of the photocurrent, we stand by our observation and additional data are plotted below. Usually under illumination photocurrent induces a change in the slope of the IV curve which results in bias dependence of the photocurrent. Here, we observe a vertical shift of the curve (see figure S 6), meaning that the photoresponse is independent of the bias. Such behavior was already observed and attributed to thermoelectric effect (M. Buscema et al. Chem. Soc. Rev. 44, 3691 (2015)). There is, in our sample, a gradient in the sheet density of MoS_2 over the graphene. This behavior can be suppressed using smaller device size (*i.e.* with device made of a single MoS_2 flake): in this case we remove the gradient of absorption since our illumination source is homogeneous at the scale of a single MoS_2 layer.

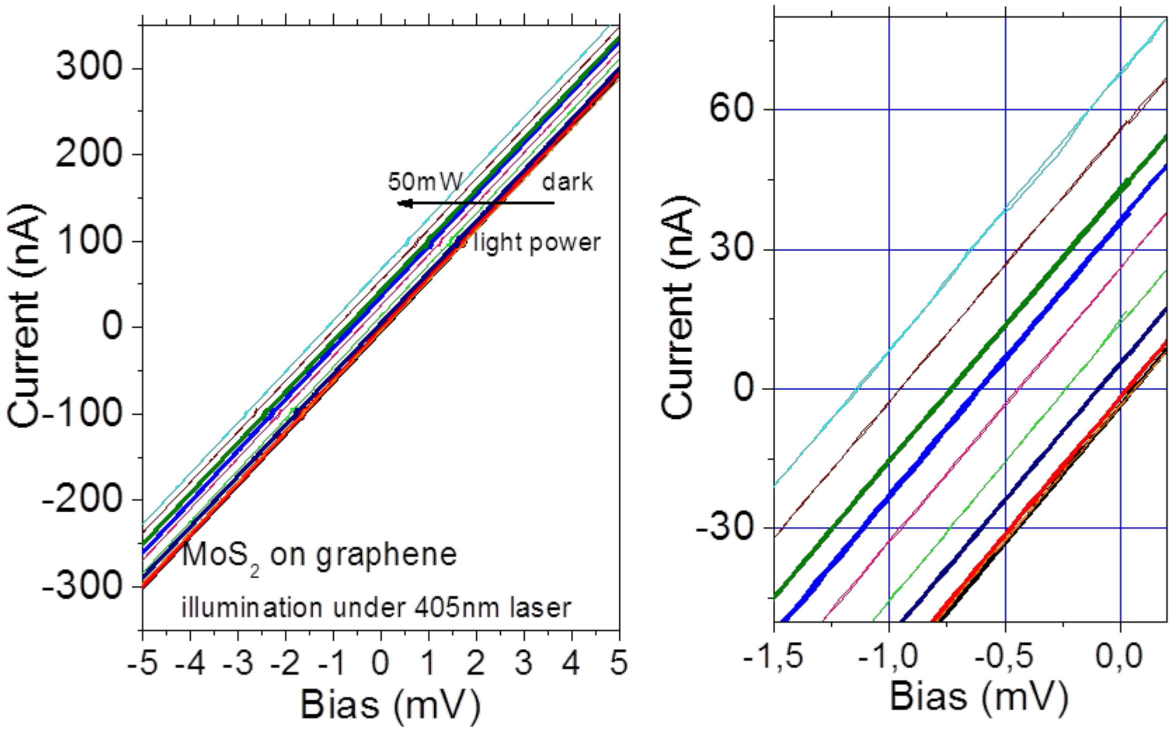

Figure S6: IV curve under different level of irradiance. The right part is a zoom of the left graph around the zero current-zero bias operating point.
